# Plasmid *parB* contributes to uropathogenic *Escherichia coli* colonization *in vivo* by acting on biofilm formation and global gene regulation

**DOI:** 10.3389/fmolb.2022.1053888

**Published:** 2022-12-16

**Authors:** Ningning Song, Henri De Greve, Quanjun Wang, Jean-Pierre Hernalsteens, Zhaoli Li

**Affiliations:** ^1^ School of Life Science and Technology, Weifang Medical University, Weifang, China; ^2^ Department of Biology, Vrije Universiteit Brussel, Brussels, Belgium; ^3^ VIB-VUB Center for Structural Biology, Vrije Universiteit Brussel, Brussels, Belgium; ^4^ Structural Biology Brussels, Vrije Universiteit Brussel, Brussels, Belgium; ^5^ SAFE Pharmaceutical Technology Co, Ltd., Beijing, China

**Keywords:** *parB*, uropathogenic *Escherichia coli*, biofilm, virulence, regulation

## Abstract

The endogenous plasmid pUTI89 harbored by the uropathogenic *Escherichia coli* (UPEC) strain UTI89 plays an important role in the acute stage of infection. The partitioning gene *parB* is important for stable inheritance of pUTI89. However, the function of partitioning genes located on the plasmid in pathogenesis of UPEC still needs to be further investigated. In the present study, we observed that disruption of the *parB* gene leads to a deficiency in biofilm formation *in vitro*. Moreover, in a mixed infection with the wild type strain and the *parB* mutant, in an ascending UTI mouse model, the mutant displayed a lower bacterial burden in the bladder and kidneys, not only at the acute infection stage but also extending to 72 hours post infection. However, in the single infection test, the reduced colonization ability of the *parB* mutant was only observed at six hpi in the bladder, but not in the kidneys. The colonization capacity *in vivo* of the *parB*-complemented strain was recovered. qRT-PCR assay suggested that ParB could be a global regulator, influencing the expression of genes located on both the endogenous plasmid and chromosome, while the gene *parA* or the operon *parAB* could not. Our study demonstrates that *parB* contributes to the virulence of UPEC by influencing biofilm formation and proposes that the *parB* gene of the endogenous plasmid could regulate gene expression globally.

## Introduction

Uropathogenic *E. coli* (UPEC), a major cause of urinary tract infections (UTI) (Emori & Gaynes, 1993), generally express virulence factors such as fimbrial adhesins and iron uptake proteins. Some UPEC strains, such as UTI89, carry a large endogenous virulence plasmid pUTI89. In a previous study, we found that interruption or deletion of *parB*, which encodes a type Ib partitioning protein, causes loss of the endogenous plasmid at a ratio of about one percent (Song et al., 2015). However, the *parA* and *parAB* mutants could harbor the plasmid rather stably. It would be interesting to further investigate the function of *parA*, *parB* and *parAB* in the virulence of UPEC and what genes on the chromosome are influenced by these mutations located on the endogenous plasmid.

Plasmids are extra-chromosomal genetic elements varying in size from several thousands to hundred thousand of base pairs. Plasmids contribute to the plasticity and rearrangement of the bacterial genome by horizontal gene transfer into plasmid-free cells of the same or different species and facilitate bacterial survival in specific niches. In addition, beneficial components on plasmids can be integrated into the bacterial chromosome. The genes on the endogenous plasmids could encode iron-uptake proteins, fimbriae, hemolysin and other factors functioning in symbiosis, pathogenesis and metabolism (Imre et al., 2006).

Most of the large plasmids are harbored in a single copy and under tight control, to avoid loading metabolic burden to the host and reduce the risk of plasmid loss (de la Cueva-Mendez and Pimentel, 2007). The partitioning system is one of the mechanisms to ensure that the plasmids can be stably maintained and inherited in the host (Yao et al., 2007; Salje et al., 2010; McVicker et al., 2019). Most of the known plasmid-encoded partitioning loci include three essential components, an ATPase-like protein (ParA/SopA/ParF or StbA/ParM), a DNA-binding protein (ParB/SopB/ParG or StbB/ParR) (Mohl et al., 2001) and one or more *cis*-acting centromere-like sites (*parS*/*sopC*/*parC*) (Barilla et al., 2007; Hanai and Arai, 2015). The former two components are encoded in one operon (Ringgaard et al., 2007). The centromere-like site can be bound by the DNA-binding protein, forming a partitioning complex. It was shown that the *parABS* partition system is responsible for chromosome and plasmid segregation in bacteria and archaea (Guilhas et al., 2020).

According to the ATPase encoded by the *parA* gene, the partitioning loci were divided into two types (Gerdes et al., 2000). Type I partitioning loci (*par1*) can be sub-classified into type Ia and type Ib, according to the size of the Walker-type ATPase. Type II partitioning loci (*par2*) are actin-like ATPases. However, two types of partitioning loci can sometimes reside on an endogenous plasmid (Ebersbach and Gerdes, 2001) such as the virulence plasmid pUTI89 of uropathogenic *E. coli* UTI89 (Chen et al., 2006), the F-like plasmid pB171 of enteropathogenic *E.coli* B171 (Tobe et al., 1999) and the IncHI plasmid R27 of *Salmonella typhimurium* (Sherburne et al., 2000; Lawley and Taylor, 2003). The comprehension of the network including ATP, DNA and the partitioning genes is still far from final and the plasmid partitioning system has been regarded as a simple model to understand the chromosomal segregation (Howard and Gerdes, 2010). Even though we have characterized the *parA/B/AB* insertional mutants of UTI89 in growth and plasmid stability *in vitro*, the effects of mutations on biofilm formation, an important concern of bacterial virulence, and the colonization in urinary tract *in vivo* still need to be further deciphered.

In this study, we removed the antibiotic resistance gene replacing the *parA*, *parB* or *parAB* from the previously constructed mutants (Song, et al., 2015) and constructed a *parB*-complemented strain by inserting the *parB* gene and its original promoter into the *nadB* gene, which is naturally inactive in UTI89 (Li et al., 2012). We observed that disruption of *parB* leads to a deficiency in biofilm formation *in vitro* and a reduction in the colonization of mice *in vivo*, while complementation of *parB in trans* restores the virulence of the mutant to the level of the wild type strain. In addition, we demonstrated that the *parB* mutation influences the expression of genes on both plasmid and chromosome. Collectively, this study demonstrates that the endogenous plasmid gene *parB* contributes to the virulence of UPEC strain UTI89 and shows that the genes are globally influenced by a *parB* mutation. This study could be helpful to further understand the function of the partitioning genes in bacterial pathogenesis and natural plasmid maintenance.

## Materials and methods

### Bacterial strains and growth conditions

The bacterial strains and plasmids used in this study are listed in Table 1. *E. coli* DH5α was used for cloning. The following antibiotics were used at the indicated concentrations: nalidixic acid, 20 μg ml^−1^; kanamycin, 25 μg ml^−1^; chloramphenicol 25 μg ml^−1^, carbenicillin 100 μg ml^−1^, except if mentioned otherwise.

**TABLE 1:** Strains and plasmids used in this study.

Strains/Plasmids	Relevant characteristics	Source/References
Strains		
UTI89	UPEC strain, an acute cystitis isolate, carries the plasmid pUTI89	Mulvey et al. (2001)
DH5α	*endA1 hsdR17supE44 thi-1 recA1 gyrA relA1* Δ(*lacIZYA-argF*)*U169 deoR Φ*80d*Lac* (Δ*lacZ*)M15	Woodcock et al. (1989)
DV7603	UTI89Nal^R^, spontaneous nalidixic acid resistant mutant of UTI89	Li et al. (2012)
DV7729	UTI89*ΔphoA*	Song et al. (2015)
DV8072	UTI89*ΔphoA traI*::miniTn*5phoA*2	Song et al. (2015)
DV8112	UTI89*ΔphoA traF*::miniTn*5phoA*2	Song et al. (2015)
DV8143	UTI89*ΔphoA p017-18*_*14645*_::miniTn*5phoA*2, IR1^a^	Song et al. (2015)
DV8147	UTI89*ΔphoA p017-18*_*14579*_::miniTn*5phoA*2, IR2^a^	Song et al. (2015)
DV8163	UTI89*ΔphoA parB*::miniTn*5phoA*2	Song et al. (2015)
DV8165	UTI89*ΔphoA traV*::miniTn*5phoA*2	Song et al. (2015)
DV9070	UTI89*ΔphoA ΔpUTI89*	This study
DV9679	UTI89 *ΔparB*::*cat*	Song et al. (2015)
DV9383	UTI89 *ΔparA*::*Km*	Song et al. (2015)
DV9384	UTI89 *ΔparAB*::*Km*	Song et al. (2015)
DV9387	UTI89 *ΔparA*	This study
DV9388	UTI89 *ΔparAB*	This study
DV9920	UTI89 *ΔparB*	This study
DV9984	UTI89 *ΔparB* *nadB*::*p060-cat*	This study
DV10143	UTI89 *ΔparB nadB*::*Pp060*(pCP20)	This study
DV10145	UTI89 *ΔparB nadB*::*p060*	This study
Plasmids		
pKD46	pINT-ts *araC*-P_*araB*_ *exo*, Cb^R^	Datsenko and Wanner, (2000)
pKD3	Cb^R^ Cm^R^ *pir* gene	Datsenko and Wanner, (2000)
pCP20	Cb^R^ Cm^R^ FLP *rep-ts*	Datsenko and Wanner, (2000)
pDONR221	Km^R^ *ccdB*	Invitrogen
pDONR221-*nadB*_U_	Km^R^, with insertion of *nadB*_UTI89_	Li et al. (2012)
pDONR221-A-cat	Km^R^ Cm^R^	This study
pDONR221-nadB_U_-catp060	Km^R^ Cm^R^	This study

^a^ , IR, intergenic region between open reading frames *p017* and *p018*.

### Plasmid construction

The PCR primers used for amplification of target fragments are listed in Table 2. The PCR reactions were performed using Ex Taq DNA polymerase (Takara Bio) at the appropriate annealing temperature. The plasmid pDONR221-*nadB*_U_-*p060*-*cat* was generated by the following steps. The *parB* with its promoter region was amplified with the primer set EcorpP060F-pP060RP1 (Table 2). The *cat* cassette was obtained with the primer set P1-EcorP2 (Table 2), using pKD3 as the template. Then overlapping PCR was performed with the mixture of above two fragments as template and the primer set EcorpP060F-EcorP2 (Table 2). The resulting fragment, as well as pDONR221-*nadB*_UTI89_, which was constructed as pDONR221-*nadB*_K12_ (Li et al., 2012), were digested with *EcoRI*. The digested products were ligated and transformed by electroporation. The positive clones were selected and then further confirmed by PCR and sequencing.

**TABLE 2:** Primers used in this study.

Name	Sequence (5′-3′)	Purposes
p060IF	AGC​TTT​ACC​CGG​TGG​TGC​ATG​T	Identification of inactivation of p060, p061 or *arAB*
p060IR	ATG​GTG​CCA​TGC​CGT​TTT​TAT​CGA	
XhoIP1	ATC​GGC​TCG​AGT​GTA​GGC​TGG​AGC​TGC​TTC	Amplifying the chloramphenicol resistance cassette from pKD3
XhoIP2	ATC​GGC​TCG​AGC​ATA​TGA​ATA​TCC​TCC​TTA	
P1	TGT​AGG​CTG​GAG​CTG​CTT​C	Amplifying the chloramphenicol resistance gene for overlapping the fragments of *parB* amplified by EcorP060 F and pP060RP1
EcoRP2	GCT​CGA​ATT​CCA​TAT​GAA​TAT​CCT​CCT​TA	
EcorpP060F	GCT​CGA​ATT​CTG​TAT​GCA​AGG​GTG​CTT​AAA​CAG	Amplifying the *parB* with its original promoter from pDONR221-B plasmid
pP060RP1	TAT​ACT​GCG​ACC​ATG​GTT​CAA​CAG​TGT​AGG​CTG​GAG​CTG​CTT​C	
nadBPF	GGG​GAC​AAG​TTT​GTA​CAA​AAA​AGC​AGG​CTAGC​AAG​GGT​TAG​AGT​GTC​T	Amplifying the linear fragments containinig *parBcat*, *nadB* and *nadB* upstream and downstream flanking sequences
nadBPR	GGG​GAC​CAC​TTT​GTA​CAA​GAA​AGC​TGG​GTGAC​CAG​AAC​TAT​TCC​GAA​G	
linadB5	TCG​GGT​GCT​GCT​GGC​ATT​CT	Identifying the insertion of *parBcat* into *nadB* on the chromosome
linadB6	TCG​GGT​GCT​GCT​GGC​ATT​CT	
LZL001f	CAA​GGG​CTA​CTC​TGA​CGA​TG	qRT-PCR for *parA*
LZL001r	GCC​TTG​CGG​ATA​AAC​CAT​AC	qRT-PCR for *parA*
LZL002f	ATG​GAA​GGT​TGA​CGG​GTT​AG	qRT-PCR for *stbA*
LZL002r	AGG​CAA​AGT​CAC​AGT​CAA​TG	qRT-PCR for *stbA*
LZL003f	GTC​TCA​CAC​TGT​TGA​TAT​TC	qRT-PCR for *UTI89_P098*
LZL003r	GGA​TTC​GTA​AGC​CAT​GAA​AG	qRT-PCR for *UTI89_P098*
LZL004f	TGT​GAT​TTG​CTC​CAG​TCT​TC	qRT-PCR for *cvpA*
LZL004r	GGG​TGT​TGG​GCG​TCT​GTT​TC	qRT-PCR for *cvpA*
LZL005f	TGC​CTG​ACG​AAT​AAG​TTG​TG	qRT-PCR for *visC*
LZL005r	CGC​TGA​TGC​TGG​CTG​GTA​TG	qRT-PCR for *visC*
LZL006f	GGC​TTC​GCT​TAC​CAC​TTT​GC	qRT-PCR for *Fic*
LZL006r	ACT​GAG​CTG​GCA​GGG​TAT​CG	qRT-PCR for *Fic*
LZL007f	TGT​CCT​GTC​ACG​ATG​GTT​TC	qRT-PCR for *BsmA*
LZL007r	GCA​AGG​GTT​ACA​GCG​AAT​AG	qRT-PCR for *BsmA*
LZL008f	GTC​TGG​GAA​CGG​ATA​AAC​TG	qRT-PCR for *actP*
LZL008r	TCT​CTA​TCA​GCG​TGG​CAT​TC	qRT-PCR for *actP*
LZL009f	AGA​TAC​CAG​TGA​GAA​CAA​AG	qRT-PCR for *YjcA*
LZL009r	CGC​ATT​TCA​GGG​AGT​TAG​TC	qRT-PCR for *YjcA*
LZL010f	TCT​CTT​TAC​GCA​CCC​AGT​TG	qRT-PCR for *Acs*
LZL010r	GAT​GAA​GAT​GGC​TAT​TAC​TG	qRT-PCR for *Acs*
LZL011f	GAT​GGG​CTG​GTC​GGT​AAA​TG	qRT-PCR for *FimH*
LZL011r	TAA​TGG​TTT​CTG​GGT​AAT​CG	qRT-PCR for *FimH*
LZL012f	ACC​TGT​TAG​ACG​CTG​ATT​AC	qRT-PCR for *gapA*
LZL012r	CAC​CAA​CTT​CGT​CCC​ATT​TC	qRT-PCR for *gapA*
LZL013f	GCG​ATT​CAC​GAC​GGC​TTT​AC	qRT-PCR for *dnaE*
LZL013r	TCA​CCC​AGA​CGC​ACA​GTT​AC	qRT-PCR for *dnaE*
LZL014f	ATT​GTG​GCG​GGT​GCT​GAA​AG	qRT-PCR for *dld*
LZL014r	CCA​GCA​CTT​GTT​CGC​CCT​TG	qRT-PCR for *dld*

### Construction of the complemented strain

Complementation of the *parB* mutant strain was realized by inserting *parB* with its promoter region into the *nadB* gene on the chromosome. The Red recombination system was employed to recombine the linear fragment amplified by the primer set nadBPF-nadBPR (Table 2) with pDONR221-*nadB*_UTI89_-*p060*-*cat* as the template. The complemented strain UTI89*Δ*p060 *nadB::p060cat* was confirmed by PCR with the primer set linadB5-linadB6 (Table 2) and sequencing.

### Motility assay

Motility assays were performed by picking fresh bacterial colonies with toothpicks onto the surface of LB plates containing 0.3% agar and incubating at 30°C for 16 h. The diameter of cloudy area of bacteria was measured. The motile capability was expressed in the cloudy area percentage of the mutants which was calculated with the formulation that the cloudy area of the wild type strain is divided by that of the transposon insertion mutant (Zdziarski et al., 2008).

### Yeast agglutination test

Briefly, agglutination of yeast cells was examined by mixing 25 μl of overnight *E. coli* culture in LB with 25 μl of a 5% commercial baker’s yeast suspension in PBS (per litre: 10 g NaCl, 0.25 g KCl, 0.1438 g Na_2_HPO_4_, 0.25 g KH_2_PO_4_; pH 7.4). Occurrence of visible clumping was compared between mutants and wild type strains (Li et al., 2012).

### Biofilm formation assay

Biofilm formation assays were carried out using crystal violet staining in 96-well microliter-plates and quantified as previously described (Merritt et al., 2005). Briefly, the bacterium was inoculated in a 5-ml minimal A medium supplemented with nicotinamide and grown to stationary phase. Then these cultures were diluted by 1:100 in minimal A medium supplemented with nicotinamide. Of each dilution 100 μl was plated in sets of three wells in a round-bottom 96 well plate (BD Falcon™ no. 353911, U bottom). The plate was covered and incubated at 30°C for 48 h. The cultures were cautiously removed using a pipette and the wells were washed twice with PBS and then stained for a conventional crystal violet assay. Briefly, biofilms were incubated in aqueous crystal violet solution (0.05% w/v) for 30 min at room temperature followed by four washes with PBS. The biofilms on the wells of the microtiter plates were photographed by a Canon digital camera. To quantify biofilm formation, 150 µl of 96% ethanol (v/v) was added to each well, incubated for 10 min at room temperature and 100 µl of the extracted solution was removed to a new 96-well plate and the optical density was determined at 595 nm.

### RNA extraction and cDNA synthesis

Total RNA was extracted from the tissue using TRIzol® Reagent according to the manufacturer’s instructions (Invitrogen) and genomic DNA was removed using DNase I (Takara). Then RNA quality was determined by 2,100 Bioanalyser (Agilent) and quantified using the ND-2000 (NanoDrop Technologies). High-quality RNA (OD_260/280_ = 1.8–2.2, OD_260/230_ ≥ 2.0, RIN ≥ 6.5, 28 S:18 S ≥ 1.0, >10 μg) was used for cDNA synthesis. First-strand cDNAs were synthesized using a SuperScript III First-Strand Synthesis SuperMix Kit (Invitrogen).

### Quantitative RT-PCR

Quantitative real-time PCR (qRT-PCR) was performed in a LightCycler480 II (Roche) with iQ™ SYBR Green Supermix (Bio-Rad). The reaction was in a total volume of 25 µl with 12.5 µl SYBR Green Supermix and 10-fold diluted cDNA as templates. The reaction conditions were as follows: 95°C for 3 min, 40 cycles at 95°C for 10 s, 55°C for 30 s, 72°C for 10 s, and one cycle at 50°C for 3 min. The assays were carried out in technical duplicates for three biological replicates with a no-template and a no-RT control. The housekeeping gene *gapA, dnaE* and *dld* were used for normalization. The relative expression folds were calculated using the 2^ΔΔCq^ method (Livak and Schmittgen, 2001).

### Ascending urinary tract infection mouse model

Bacteria for inoculation were grown statically for 24 h at 37°C in LB broth, collected and adjusted to OD_660_ = 1.5 in PBS (per litre: 10 g NaCl, 0.25 g KCl, 0.1438 g Na_2_HPO_4_, 0.25 g KH_2_PO_4_; pH 7.4). Eight-week-old female C3H/HeN mice (Harlan, Horst, Netherlands) were anesthetized by Anesketin (Eurovet, Brussels, Belgium) and Rompun (Bayer, Brussels, Belgium) (15 µl of each for one mouse) and infected *via* transurethral catheterization of 50 µl of bacterial suspension with 10^7^ CFU of a mixture of equal amount of the transposon insertion mutants, specific null mutants or complemented strain and UTI89Nal^R^, which is a spontaneous nalidixic acid resistant mutant of UTI89. At each indicated time point, mice were sacrificed by inhalation of carbon dioxide. The bladders and kidneys were immediately harvested and homogenized using a 5-ml vessel grinder and plain plunger. Serial dilutions were plated onto LB agar, with the appropriate antibiotics when necessary and the colony forming units (CFU) were enumerated.

### Statistical method

The Mann-Whitney test was applied for the comparison of the data collected from the single strain assay and the one-tailed paired *t*-test for the comparison of the data collected from the mixed infection assay. Both analyses were performed using GraphPad software.

## Ethical issues

The animal experiments were approved by the Ethical Committee for Animal Experiments of Vrije Universiteit Brussel (project number 06-219-3) and complied with all relevant national legislation and institutional policies.

## Results

### Insertion in *parB* and *traV* reduces biofilm formation of UTI89

**FIGURE 1:**
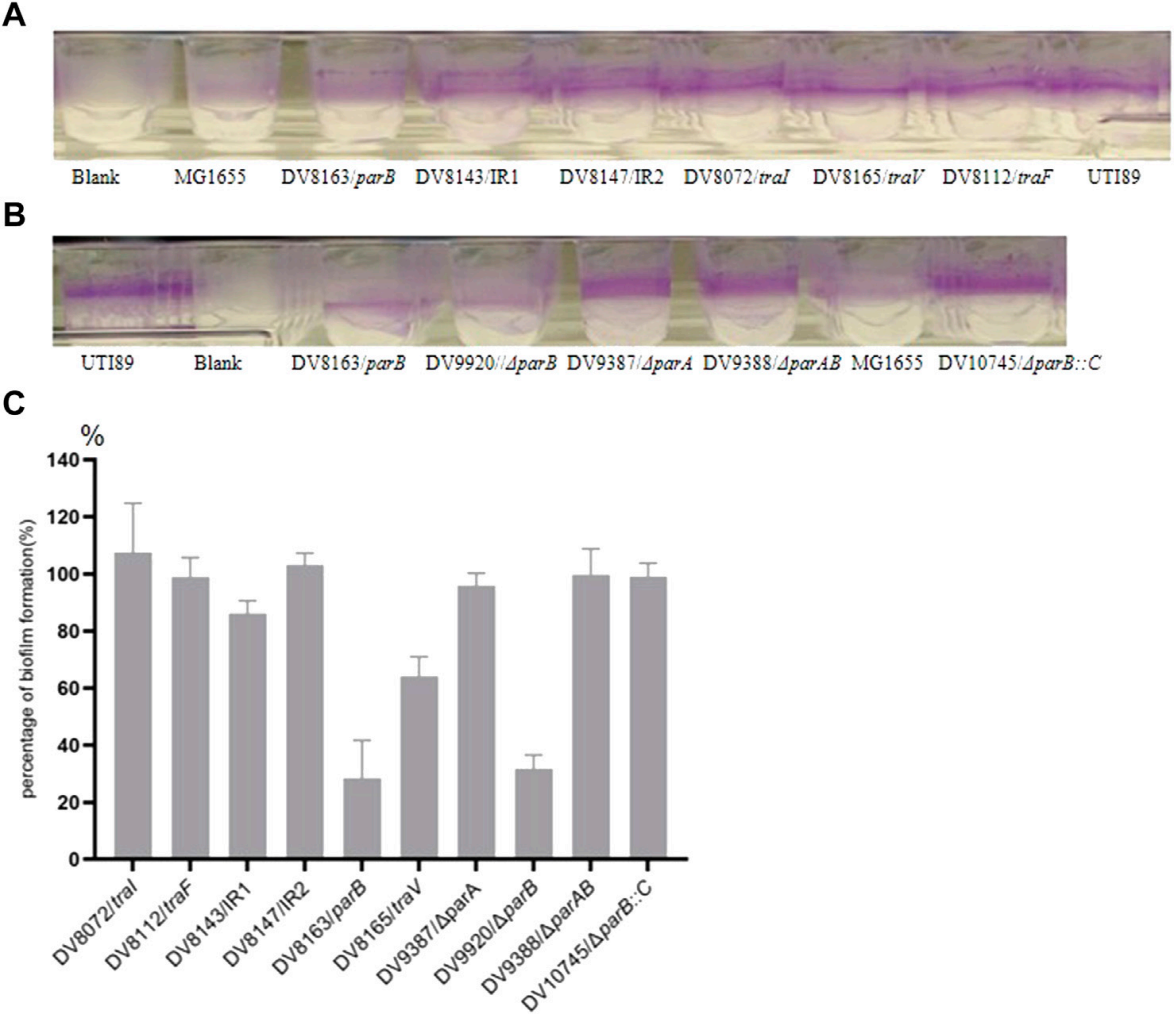
Biofilm formation by the transposon insertion mutants. **(A)** and **(B)** the biofilm formation on the wells of microtiter plates; **(C)**. Percentage of Biofilm formation by the mutants. The percentage of biofilm formation by each mutant was calculated according to the formula, [(OD_mutant_-OD_blank_)/(OD_parent strain_-OD_blank_)]×100%. The mutants with the disrupted genes are indicated in the X-coordinate. IR = intergenic region between *p017-18* open reading frames.

In a previous study, we isolated several transposon miniTn*5phoA2* (Pattery et al., 1999) insertion mutants with the insertion localized on the endogenous plasmid (Song, et al., 2015). In the present investigation on the influence of these mutations on the *in vitro* phenotype of UTI89, we discovered that mutant DV8163 and DV8165 (*tra*V) show a relatively low biofilm formation on the flexible plates among the six mutants derived from DV7729 with insertions in the plasmid (Song, et al., 2015). DV8163 only formed around 20% biofilm and DV8165 around 60% (Figure 1). DV8163 harbors miniTn*5phoA*2 fused into the *parB* ORF at the end of the *parB* gene as described previously (Song et al., 2015). The *parB* gene is the downstream gene within the *parAB* operon in pUTI89, encoding a DNA-binding protein. The specific parB/p060 knock-out strain DV9920 demonstrated the same biofilm formation as the transposon insertion mutant DV8163 (Figure 1). The complemented strain DV10145, which was consrtucted by reintroduction of *parB* with its natural promoter sequence into the naturally inactivated *nadB* of DV9920, recoved the biofilm formation to the level of the wild type strain (Figure 1).

**FIGURE 2:**
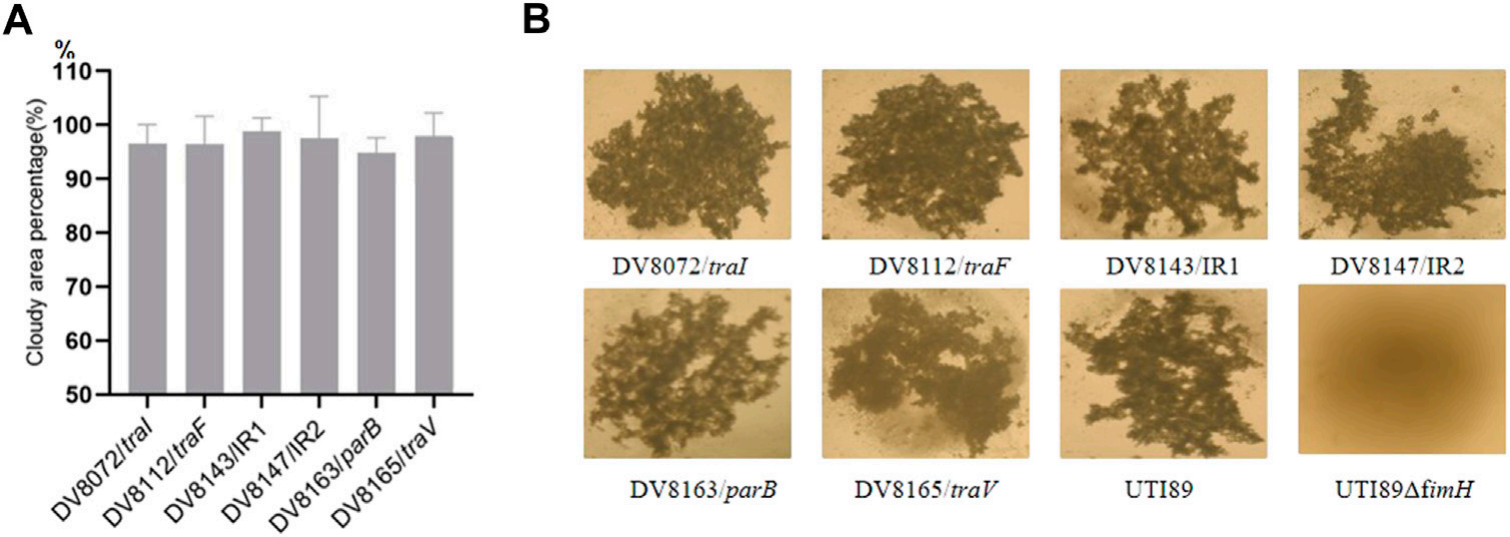
Motility and yeast agglutination assay of transponson insertional mutants. **(A)**. The percentage of cloudy area formed by the transposon insertional mutants comparing to that formed by the wild strain. **(B)**. Yeast agglutination by the transposon insertional mutants with the wild type strain as a positive control and *fimH* deletion strain as a negative control. IR = intergenic region between *p017-18* open reading frames.

The mutant DV8165 carries the miniTn*5phoA*2 insertion at the beginning of the *traV* gene. The *traV* gene encodes an outer membrane lipoprotein (Harris et al., 2001), which functions in the assembly of F-type pili. All mutants derived from DV7729 have similar motility and agglutinate yeast cells as UTI89 (Figure 2), including the other two mutants and DV8072 with insertion in *traI* and DV8112 with the insertion in *traF*. Both DV8143 and DV8147 harboring the insertion in the intergenic region between the UTI89_*p01*7 and UTI89_*p018* at different nucleotide positions have the same phenotypes as UTI89 in the above test.

### Reduced colonization of the *parB* transposon insertion mutant in mice

**FIGURE 3:**
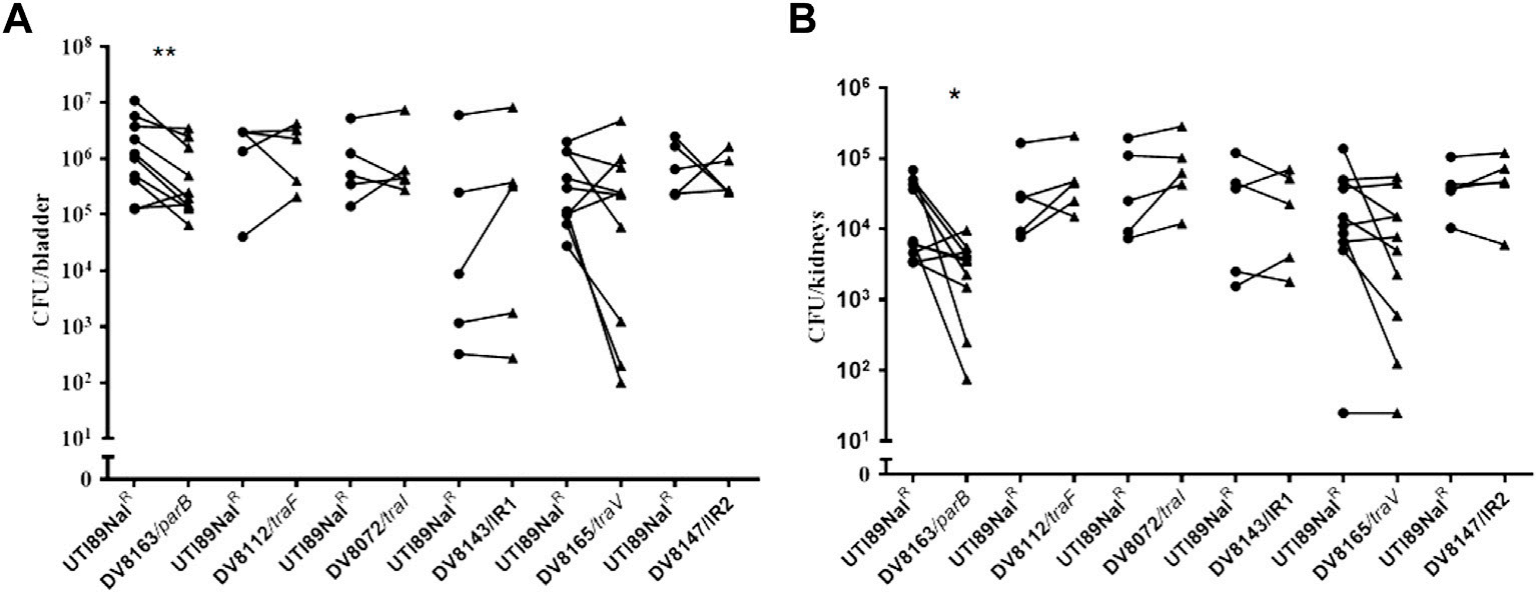
*In vivo* infection of mice with mutant strains. Mixed colonization of mutants and UTI89Nal^R^ in bladder **(A)** and kidneys **(B)** of mice. The mice were sacrificed at 24 h post infection. *, *p* < 0.05; **, *p* < 0.01. Circles stand for the wild type strain and the triangles represent the transposon insertion mutants, which are labeled on the *X*-axis respectively. One symbol represents the number of the bacteria recovered from the bladder or kidneys from one mouse. IR = intergenic region between *p017-18* open reading frames.

Since endogenous plasmids have been regarded as virulence factors in bacterial pathogens, we further investigated the consequences of these pUTI89 insertions on the bacterial colonization in the well-established ascending infection mouse model by mixed bacterial challenge. The bacterial burden in the bladder and kidneys of C3H/HeN mice was investigated at 24 h post infection. In the mixed infection, the bacterial burden of DV8163, a miniTn*5phoA2* insertion in the *parB* gene, in the bladder is reduced in seven out of ten mice; there is no obvious difference in the other three mice. The bacterial load of DV8163 in the kidneys is reduced in eight out of ten mice and in the other two mice the load is slightly increased (Figure 3). Furthermore, comparing the number of bacterial colonies from the mice infected by mutant DV8163 and wild type strain demonstrated that the mutant DV8163 has a significantly lower bacterial burden than the wild type strain in the bladders (*p* = 0.0040) and kidneys (*p* = 0.0104) in mixed infection (Figure 3).

No significant difference was detected with any of the other five pUTI89 mutants, including mutants DV8072 (*traI*), DV8112 (*traF*), DV8165 (*traV*), DV8143 and DV8147, in the bladder and kidneys of mice at 24 h post infection (Figure 3). The results from the mutants DV8143 and DV8147 are consistent with the single *in vivo* colonization test of a *p017-18* mutant reported before, even though the previous test was done at six hpi (Cusumano et al., 2010).

We also tested whether plasmid-free UTI89 clones can be recovered from the mice infected with the *parB* mutant DV8163, which generates one percent plasmid-free bacteria *in vitro* (Song et al., 2015). The suspension of the grinded bladders or kidneys of mice was spread on LB agar plates without kanamycin and incubated overnight at 37°C. Then the resulting bacterial colonies were replica plated using sterile velvet to LB agar plates with kanamycin. All the tested colonies grew well, implying that all recovered bacteria colonizing the urinary tract of the mice harbored the plasmid. Given that the plasmid can be lost at a ratio of one percent *in vitro*, we presume that an unknown selection mechanism is exerting a pressure to retain the endogenous plasmid *in vivo*.

### Deletion of *parB* causes colonization deficiency of UTI89

**FIGURE 4:**
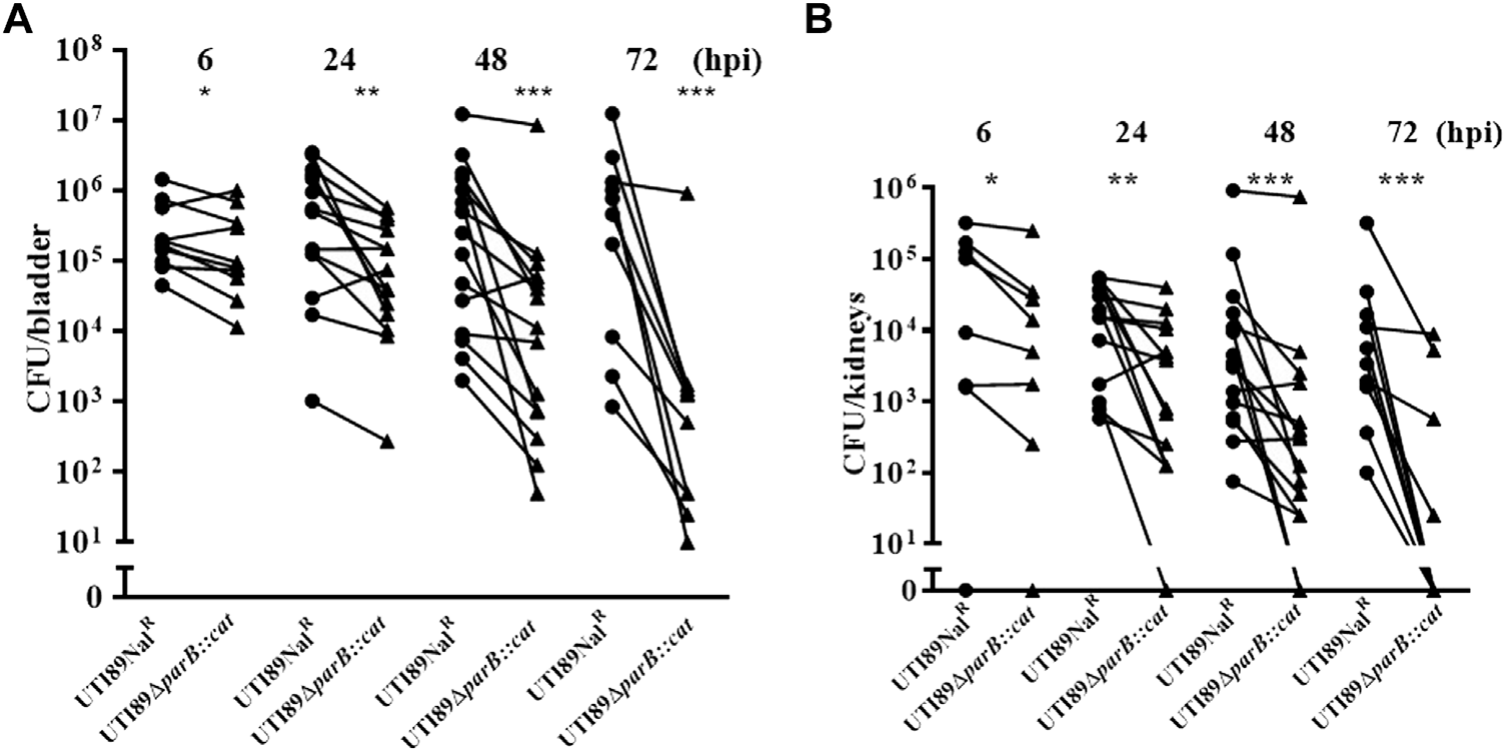
Mixed infection of mice with equal amounts of the *parB* mutant and the wild type strain UTI89Nal^R^. UTI89*ΔparB*:*cat* instead of DV9679 was used for simple demonstration the colonization in bladder **(A)** and kidneys **(B)** with the deleted genes. *, *p* < 0.05; **, *p* < 0.01; ***, *p* < 0.001. Circles stand for the wild type strain and the triangles represent the transposon insertional mutants, which are labeled on the *X*-axis, respectively. One symbol represents the number of the bacteria recovered from the bladder or kidneys from one mouse.

Since the *parB*::miniTn*5phoA2* mutant DV8163 showed decreased colonization ability *in vivo*, we investigated whether the transposon insertion mutant of *parB* as well as the *parA* and *parAB* deletion mutants, alone or mixed with the wild type strain, is attenuated in the mouse model. Interestingly, we found that a statistically significant difference between DV9679 (UTI89 *ΔparB::cat*) and the DV7603 strain, a spontaneous nalidixic acid resistant mutant of UTI89, has emerged at six hpi both in the colonization in the bladder and kidneys and the difference broadened further with the elongation of the co-inoculation time span to 24 and 72 hpi. Even though the major trend is that the DV7603 strain outnumbers the mutant DV9679 at the first three time points, the mutant DV9679 occasionally colonizes slightly more efficiently than the wild type strain in the bladder and kidneys of one to three of the ten mice tested in each group (Figures 4A,B). The mutant DV9679 is outnumbered by the DV7603 strain in the bladder and kidneys of all 10 mice tested at 72 hpi (Figures 4A,B).

Unexpectedly, no statistically significant difference can be detected between the *parA* mutant DV9383 or the *parAB* mutant DV9384 and the DV7603 strain either in bladders or in kidneys of the tested mice at any time after infection, including six hpi, 24 hpi, 48 hpi and 72 hpi (Supplementary Figure S1). In conclusion, deletion of *parB* reduces colonization ability compared to DV7603, but deletion of *parA* or *parAB* does not lead to a decrease in colonization of murine bladder or kidneys.

**FIGURE 5:**
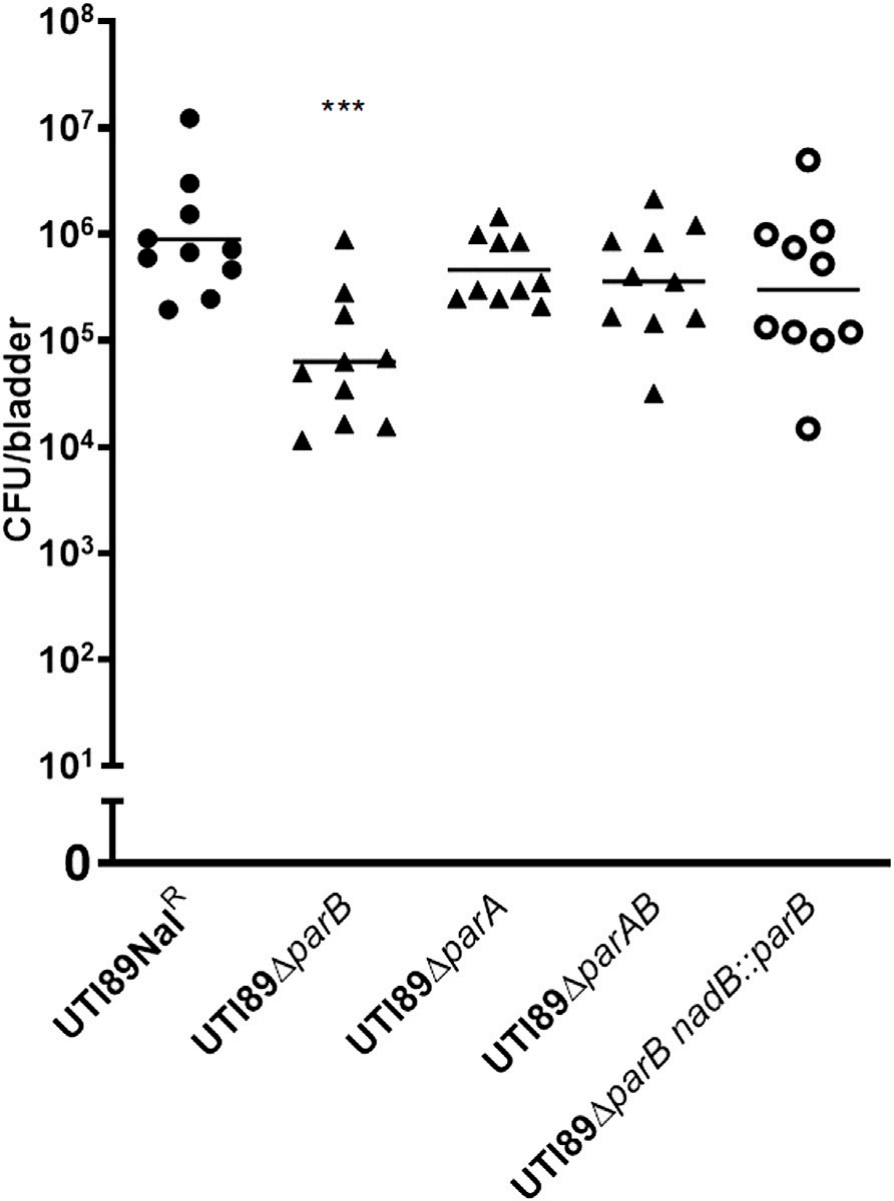
Single colonization tests *in vivo*. The lines in each column indicate average CFUs of bacteria recovered from the bladder. The mice were sacrificed at 6 h hours post infection as mentioned. The filled dots represent the wild type strain, the filled triangles represent the gene deletion mutants and the hollow circle represent the *parB-*complemented strain, which are labeled on the *X*-axis, respectively. One symbol represents the number of the bacteria recovered from the bladder or kidneys from one mouse. ***, *p* < 0.001.

We tested whether the single colonization would show similar differences as in the mixed incubation test in mice. Mice infected with the DV7603 strain or the mutants DV9679, DV9383 or DV9384 were sacrificed at 6, 24, 48 and 72 hpi and the number of CFU recovered from bladder and kidneys was counted. The colonization ability of mutant strains DV9383 and DV9384, with deletions of the *parA* or *parAB* genes respectively, was not statistically significantly reduced in the bladder and kidneys at these tested time points (Supplementary Figure S2), compared to the DV7603 strain. In contrast, the mutant DV9679 with a deletion of the *parB* gene displayed a reduced colonization at six hpi in the bladder (Figure 5), but not at the other three time points, and not in kidneys at all four tested time points (Supplementary Figure S2).

### Complementation of the *parB* mutant restores the colonization ability in mice

**FIGURE 6:**
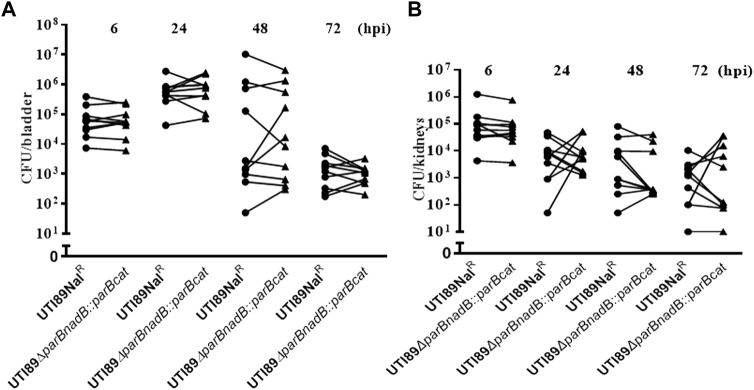
The colonization ability of *parB*-complemented and wild type UTI89 strains in an UTI mouse model. **(A)** In bladder of mice. **(B)** In the kidneys of mice. Circles stand for the wild type strain and the triangles represent the *parB*-complemented strain, which are labeled on the *X*-axis. One symbol represents the number of the bacteria recovered from the bladder or kidneys from one mouse.

To exclude that the above observation would be caused by a polar effect of the *parB* mutation on the nearby genes and to confirm that the *parB* gene is a virulence factor following Koch’s postulates, we introduced the *parB* gene with its promoter region into the *nadB* gene region on the UTI89 chromosome and performed transurethral inoculation into the bladder of mice with this complemented strain DV9984, alone or together with an equal number of DV7603. The colonization by the mixture was assessed at six hpi, 24 hpi, 48 hpi and 72 hpi, both in bladder and kidneys of ten mice in each group (Figure 6). No statistically significant differences were detected at the four time points (Figure 6). The colonization ability in the urinary tract by DV9984 alone is recovered also in the bladder of mice (Figure 5). This *in vivo* test demonstrated that the *parB* gene inserted in the chromosome can complement the attenuated colonization ability in the bladder and kidneys caused by the deletion of the *parB* gene on the plasmid.

### qRT-PCR confirms the influence of deletion of *parB* on gene expression

**FIGURE 7:**
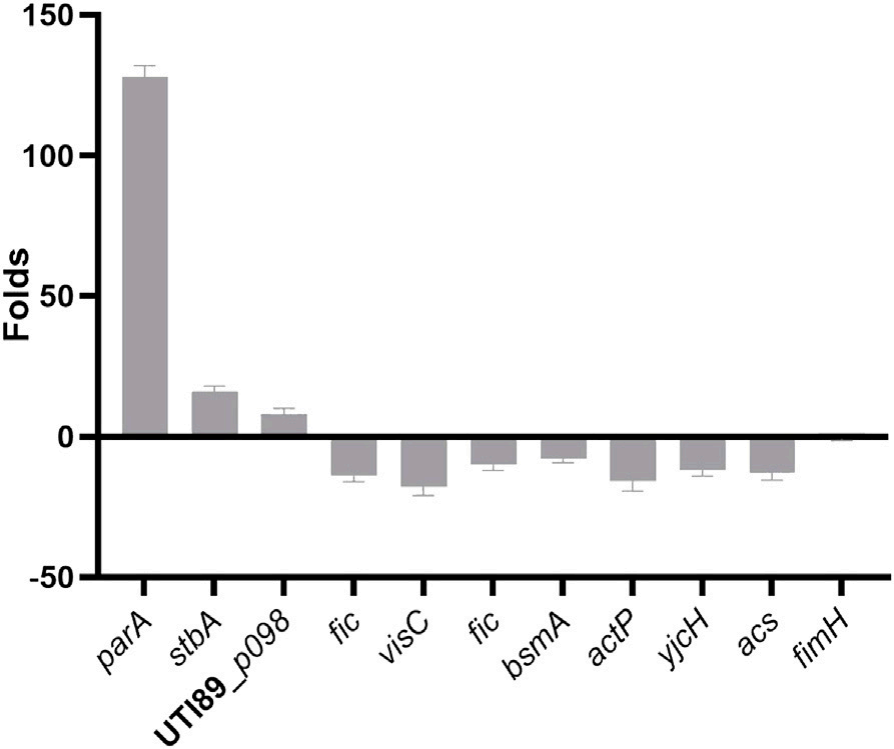
Relative expression of virulence genes in UTI89Δ*parB* compared to UTI89.

To further illustrate the effects of the *parB* mutation on the transcription of virulence-related genes of UTI89, RNA was extracted and expression levels were analyzed by qRT-PCR, comparing the UTI89*ΔparB* mutant with UTI89 at the exponential growth stage. The expression level variation between in the mutant UTI89*ΔparB::cat* and the wild type strain is shown in Figure 7.

We mainly focused on plasmid genes involved in plasmid stability, separation and stable inheritance and some virulence or virulence-related genes on the chromosome. Deletion of *parB* (UTI89*_p060*) causes the expression of *parA*, *p062*(*stbA*) encoding a plasmid segregation protein, UTI89*_p098* encoding type I toxin-antitoxin system Hok family toxin increased 128, 16 and eight folds respectively. The changed expression of *stbA* by the deletion of *parB* is consistent with the previous report in the mini plasmid model (Gerdes et al., 2000).

Deletion of *parB*, also causes changes in gene expression on the chromosome. As a binding protein, ParB possibly binds to the regulatory regions of the affected genes or indirectly affects gene expression by influencing chromosomal genes. The gene *cvpA* (UTI89*_C2597*) (Shaffer et al., 2017) and *visC* (UTI89*_C3292*) (Floyd et al., 2016) were chosen since they are involved in biofilm formation of UPEC. Indeed, the expression level of *cvpA* and *visC* in the *parB* mutant is down-regulated 10–20 fold compared to the wild type strain. *Fic* (UTI89*_C3864*) encoding a cell filamentation protein involved in the synthesis of PAB or folate decreased 10-fold in the *parB-*deleted strain. The Fic protein was shown to be essential for growth of *E. coli* and involved in regulation of cell division (Komano et al., 1991). *BsmA* (UTI89*_C4789*) encoding biofilm stress and motility protein decreased 8-fold in the *parB* deleted strain. The *bsmA* mutants are altered in their ability to produce biofilms, in biofilm structure, in cell motility, and in response to pH and oxidative stress (Weber et al., 2010). *ActP* (UTI89*_C4657*) encoding acetate permease, *YjcH* (UTI89*_C4658*) encoding inner membrane protein and *ACS* (UTI89*_C4659*) encoding acetyl-CoA synthetase decreased nearly 16 folds in the *parB*-deleted strain. It was shown that deletion of the *acs-yjcH-actP* operon in *E. coli* not only decreases its cytotoxic level to macrophages, but also attenuates virulence and colonization capability in avian lungs *in vivo* for colibacillosis infection models (Zhuge et al., 2019). However, as a main virulence factors of UPEC, the expression of *FimH* (UTI89*_C5017*), the type-1 fimbrial tip adhesin, is not influenced significantly by mutation of *parB*.

## Discussion

We systematically tested virulence contribution of the *parAB* operon and the single genes *parA* and *parB* by comparing the virulence of the mutants with the wild type strain. The *parB* gene is especially important for the bacterial colonization. It was reported that *parAB* enhances the stability of a mini-R plasmid more than *stbAB* does (Ebersbach and Gerdes, 2001). In our previous study, we confirmed that deletion of *parB* could cause endogenous plasmid loss (Song, et al., 2015). However, the mutation did not influence the growth of the mutant strain without antibiotic pressure (Song, et al., 2015). To our knowledge, no report has been presented yet about the function of the component genes in the partitioning operon in the pathogenesis of uropathogenic *E. coli*. Here, we report that the *parB* gene contributes to virulence of UPEC in a mouse model potentially through biofilm formation and global regulation of gene expression.

Although several mutants (*traV*, *traI* and *traF*) involved in pilus assembly were tested, only the *traV* mutation shows a reduction of biofilm formation up to 60% compared to the parental strain (Figure 1). This could result from either the subcellular location of the gene products or the potential polar effects of the transposon insertion into *traV* (DV8165), since many genes involved in pilus assembly are located downstream of the insertion. However, the colonization ability of the *traV* mutant in the mouse model is equal to the wild type strain.

These data on the colonization of the *parB* mutant *in vivo* in single strain infection in our study are consistent with a previous investigation, using plasmid-free cells or deletion mutants of other potential virulence genes on the plasmid (Cusumano et al., 2010). In that report, the plasmid-free bacterium was isolated by treatment with ethidium bromide and also carried a *stbAB* deletion. The colonization ability of this plasmid-free UTI89 was greatly reduced *in vivo* (Cusumano et al., 2010). However, in our study, we did not observe the same reduction in the bladder or kidneys with the plasmid-free strain isolated without chemical treatment and not carrying the *stbAB* deletion (Supplementary Figure S3). Both studies confirmed that some of the plasmid components (*p028-p030* and *p062-p063* in the previous study) including the *parB* gene are important factors for virulence in the acute colonization stage in an UTI mouse model. However, in our study the *parAB* (*p060-p061*) mutant (Supplementary material Figures S1, S2) displays a different colonization behavior from the *p062-p063* (*stbAB*) deletion mutant (Cusumano et al., 2010), considering that both operons function in plasmid maintenance and stability. Combination of our results and the previous study that deletion of *parAB* causes the plasmids to be less stable than deletion of *p062-p063* (*stbAB*) when the corresponding fragments were inserted in a mini-R plasmid (Ebersbach and Gerdes, 2001) suggests that the *parAB* system is not only crucial for stable plasmid inheritance, but also would have an important function *in vivo* colonization. So, it is reasonable to declare that the *parB* gene of the *parAB* operon plays a role in the virulence of UPEC, both in biofilm formation *in vitro* and in colonization in mouse.

We used qRT-PCR to investigate the impact of the *parB* deletion on genes encoding known virulence factors, the proteins contributing to plasmid stability, and the component essential for biofilm formation. The expression of the adhesin FimH, one of the most important virulence factors of UTI89 is not influenced by the *parB* mutation. Genes relevant to plasmid stability, such as *parA* and *stbAB* are upregulated, which means that these genes could be in a fluctuating way expressed and accelerate plasmid instability when *parB* is absent. The gene *parA* product could accumulate in bacterial cell since it cannot be recruited to *parB-parS* complex to function in proper plasmid segregation. Indeed, the interaction of the ParA and ParB components, which leads to proper separation of plasmid copies, has been extensively studied in plasmids and bacteria of the proteobacteria phylum. These studies provide the foundation for filament- and non-filament-based models of plasmid and bacterial chromosome segregation (Lioy et al., 2015). Indeed, a recent study shows that ParB binds CTP and hydrolyzes it upon interaction with centromer-like *parS* motifs (Osorio-Valeriano et al., 2019). The CTPase activity of ParB is critical for the partition complex formation *in vivo* and potentially it could mediate a regulatory link between CTP-dependent metabolic pathways and DNA segregation (Taylor et al., 2021).

Taken together, our study presented here determined that *parB* is also involved in the virulence of UPEC, and not only in plasmid segregation and potentially involved in the regulation of gene expression in UPEC. Our results shed light on the contribution of the partitioning gene *parB* to the virulence and gene regulation of UPEC and identify novel virulence genes.

## Data Availability

The original contributions presented in the study are included in the article/Supplementary Material, further inquiries can be directed to the corresponding author.

## Supplementary material

SUPPLEMENTARY FIGURE S1 Mixed infection test of the *parA* or *parAB* mutant with the wild type strain UTI89Nal^R^. UTI89*ΔparA*::*Km* instead of DV9383, UTI89*ΔparAB*::*Km* instead of DV9384 are used for simple demonstration the colonization with the deleted genes. No significant differences were observed either in bladder or in kidneys by the *parA* or *parAB* mutant comparing to the wild type strain. Circles stands for the wild type strain and the triangles represent the *parB*-complemented strain, which are labeled on the X-axis. One symbol represents the number of the bacteria recovered from the bladder or kidneys from one mouse.

SUPPLEMENTARY FIGURE S2 Single colonization tests *in vivo*. Horizontal lines in each column indicate average CFUs of bacteria recovered from the bladder **(A)** or kidneys **(B)**. The mice were sacrificed at the indicated time points post infection as mentioned in the figure. Circles standfor the wild type strain and the triangles represent the transposon insertion mutants, which are labeled on the X-axis respectively. One symbol represents the number of the bacteria recovered from the bladder or kidneys from one mouse.

SUPPLEMENTARY FIGURE S3 Single colonization tests of UTI89 and UTI89 plasmid-free strain *in vivo*. No differences were observed in colonization capacity between two strains in bladder and kidneys of the infected mice. Circles stand for the wild type strain and the squares represents the plasmidfree UTI89, which are labeled on the X-axis respectively. One symbol represents the number of the bacteria recovered from the bladder or kidneys from one mouse.
