# Supplementary figures and images for: Plasmid parB contributes to uropathogenic Escherichia coli colonization in vivo by acting on biofilm formation and global gene regulation
Source: Front Mol Biosci. 2022 Dec 16;9:1053888. doi: 10.3389/fmolb.2022.1053888 (PMC9800825; doi:10.3389/fmolb.2022.1053888)

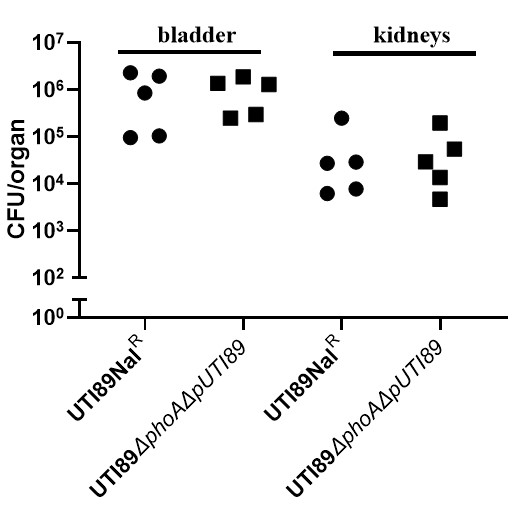

Supplement: Supplementary file 1 [file Image3.JPEG]

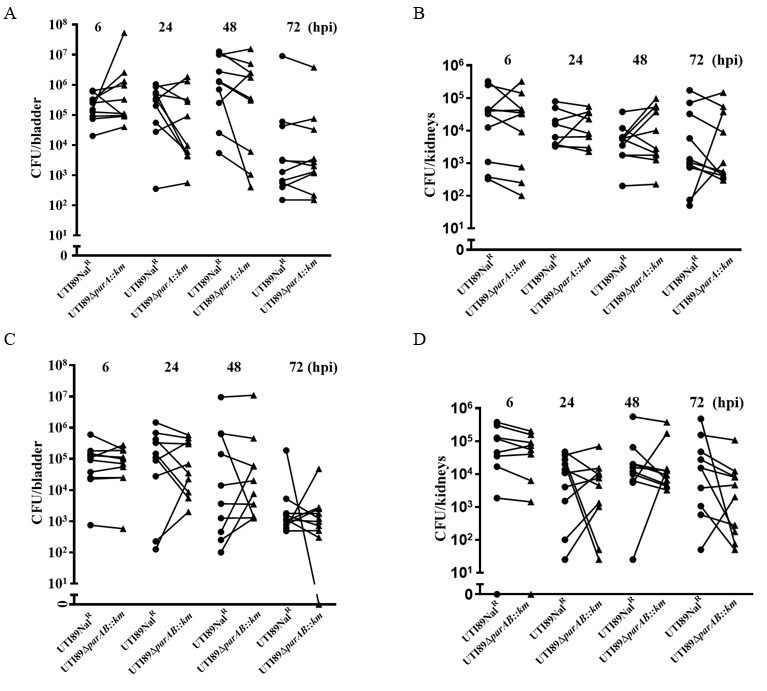

Supplement: Supplementary file 2 [file Image1.JPEG]

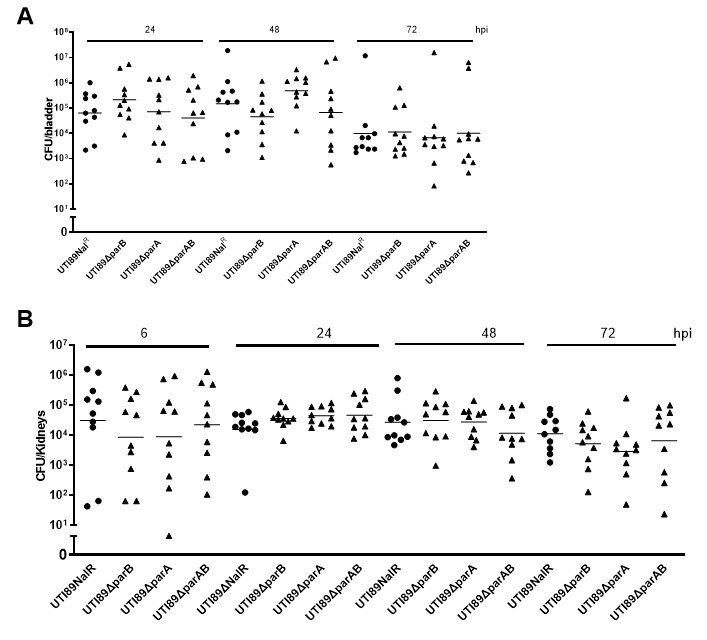

Supplement: Supplementary file 3 [file Image2.JPEG]
